# Predictive factors of clinical success after adrenalectomy in primary aldosteronism: A systematic review and meta-analysis

**DOI:** 10.3389/fendo.2022.925591

**Published:** 2022-08-18

**Authors:** Worapaka Manosroi, Pichitchai Atthakomol, Phichayut Phinyo, Piti Inthaphan

**Affiliations:** ^1^ Division of Endocrinology, Department of Internal Medicine, Faculty of Medicine, Chiang Mai University, Chiang Mai, Thailand; ^2^ Orthopaedics Department, Faculty of Medicine, Chiang Mai University, Chiang Mai, Thailand; ^3^ Clinical Epidemiology and Clinical Statistic Center, Faculty of Medicine, Chiang Mai University, Chiang Mai, Thailand; ^4^ Department of Family Medicine, Faculty of Medicine, Chiang Mai University, Chiang Mai, Thailand; ^5^ Department of Internal Medicine, Nakornping Hospital, Chiang Mai, Thailand

**Keywords:** primary aldosteronism, adrenalectomy, clinical success, predictive factors, meta-analysis

## Abstract

**Background:**

Unilateral adrenalectomy is the mainstay treatment for unilateral primary aldosteronism (PA). This meta-analysis aimed to systematically analyse predictors of clinical success after unilateral adrenalectomy in PA.

**Methods:**

A search was performed using *PubMed/Medline*, *Scopus*, *Embase* and *Web of Science* from their inception to February 2022. Observational studies in adult PA patients which reported predictors of clinical success after unilateral adrenalectomy were included. A random-effects model was employed to pool the fully adjusted odds ratio (OR) or standardized mean difference (SMD) with 95% confidence interval (95% CI).

**Results:**

Thirty-two studies involving 5,601 patients were included. Females had a higher clinical success rate (OR 2.81; 95% CI 2.06–3.83). Older patients, patients with a longer duration of hypertension and those taking a higher number of antihypertensive medications had lower clinical success rates (OR 0.97; 95% CI 0.94–0.99, OR 0.92; 95% CI 0.88–0.96 and OR 0.44; 95% CI 0.29–0.67, respectively). Compared to non-clinical success cases, patients with clinical success had a lower body mass index (SMD -0.49 kg/m^2^; 95% CI -0.58,-0.39), lower systolic (SMD -0.37 mmHg; 95% CI -0.56,-0.18) and diastolic blood pressure (SMD -0.19 mmHg; 95% CI -0.33,-0.06), lower serum potassium (SMD -0.16 mEq/L; 95% CI -0.28,-0.04), higher eGFR (SMD 0.51 mL/min/1.73m^2^; 95% CI 0.16,0.87), a lower incidence of dyslipidemia (OR 0.29; 95% CI 0.15–0.58) and a lower incidence of diabetes mellitus (OR 0.36; 95% CI 0.22–0.59).

**Conclusions:**

Multiple predictors of clinical success after unilateral adrenalectomy in PA were identified which can help improve the quality of care for PA patients.

**Systematic Review Registration:** INPLASY, identifier 202240129.

## Introduction

Primary aldosteronism (PA) is the most common cause of endocrine hypertension with a prevalence of 60% in resistant hypertension patients (1). An increased risk of cardiovascular and cerebrovascular diseases was observed in PA patients compared to essential hypertension patients (2). The goal of PA treatment includes blood pressure control, normalized serum potassium level without potassium supplements and prevention of further cardiovascular and renal complications. Currently, there are two therapeutic approaches for PA: unilateral adrenalectomy for unilateral disease and targeted medical therapy for bilateral disease (3). Surgical management by unilateral adrenalectomy in PA can reduce cardiovascular risk, improve quality of life and has the long-term advantage of greater cost-saving over life-long medical therapy (4–6). The clinical cure rate in PA after adrenal venous sampling (AVS)-guided adrenalectomy was significantly higher than in non-AVS-guided adrenalectomy (40% versus 30.5%, p=0.027) (7). Whether the operation was AVS-guided or not, the clinical cure rate was approximately 27.1% (8).

There are multiple predictive factors related to clinical success after adrenalectomy in PA. In AVS-guided adrenalectomy, duration of hypertension, gender, antihypertensive medication dosage, body mass index, target organ damage, and size of the largest nodule at imaging can help predict the clinical success after surgery in PA (9). Another study showed that gender, body mass index, duration of hypertension, creatinine levels, and number of antihypertensive medications could facilitate the prediction of clinical success after adrenalectomy (10). Different definitions of clinical cure in PA were used in each of the studies, e.g., the primary aldosteronism surgical outcome criteria (PASO) and other criteria such as normotension without the help of antihypertensive medications (11, 12). Moreover, studies comparing AVS-guided and non-AVS-guided surgery used diverse measures to diagnose unilateral PA. As AVS may play a major role in the clinical success rate after adrenalectomy, this diversity could affect clinical outcomes.

Reports of multiple predictors of clinical success after adrenalectomy in PA have been published. However, those results remain unclear and inconsistent, with varying quality across the studies. The present systematic review and meta-analysis aimed to clarify predictors of clinical success after unilateral adrenalectomy. In addition, subgroup analysis of patients with AVS-guided surgery and those without AVS-guided surgery was also conducted.

## Materials and methods

### Search strategy and selection criteria

The reporting in this study followed the Preferred Reporting Items for Systematic Reviews and Meta-analyses (PRISMA) guidelines (13). The pre-defined protocol was registered in INPLASY 202240129. A comprehensive search of four databases, *PubMed/Medline*, *Scopus*, *Embase* and *Web of Science*, was performed from their inception to February 2022. The keywords included were “hyperaldosteronism OR primary aldosteronism OR primary hyperaldosteronism OR aldosteronism” AND ”adrenalectomy OR surgical OR surgery OR unilateral adrenalectomy” AND ”patient outcome assessment OR clinical outcome OR outcome OR predictor OR predictive factor”. Medical subject heading (MeSH) terms were employed in the *PubMed*/*Medline* search. Details of the search strategy are presented in the **Supplementary Appendix**. Manual searches were conducted to identify references from the included studies, other relevant publications, and non-included reviews, and these were included as additional studies for the initial screening. Rayyan, a web-based program (Rayyan Systems Inc., Cambridge, MA, USA) (14), was employed for duplicate removal and initial screening of abstracts and titles.

Two authors (WM, PA) independently conducted the searches, screened for titles and abstracts. Pertinent studies were retrieved and underwent full-text screening for inclusion criteria. Then the two authors independently evaluated the methodological quality of the included studies and conducted the data extraction. The third author (PI) together with the first two authors (WM, PA) discussed and reached a consensus in cases of disagreement during the article search and selection processes.

Inclusion criteria for articles were as follows: 1) observational (non-randomized) studies that included adult PA patients; 2) studies that reported either predictive factors of complete clinical success versus partial success plus no clinical success as well as those that reported predictive factors for complete plus partial clinical success versus no clinical success after unilateral adrenalectomy. The predictive factors could be reported as either adjusted or unadjusted odds ratio (OR) or as crude data; 3) studies that reported the number of patients with both complete clinical success and those with partial or no clinical success after unilateral adrenalectomy; 4) standard diagnostic and/or confirmation criteria was employed to diagnose and confirm PA (15), 5) the definitions of clinical success or non-success should be clearly specified in the articles, and 6) the studies should provide adequate information in accordance with the Strengthening the Reporting of Observational Studies in Epidemiology (STROBE) statement (16). As various definitions of clinical success were used among the studies, three categories of clinical success (complete, partial and no clinical success) were grouped. Complete clinical success was defined as normal blood pressure without the use of anti-hypertensive medications. Partial clinical success was defined as less anti-hypertensive medications or a reduction in blood pressure with either the same amount or less of anti-hypertensive medications used. No clinical success was defined as unchanged blood pressure with the same amount of anti-hypertensive medications used.

In cases of duplicate studies of the same patient population, the study reporting higher number of participants was selected as the main data source. Exclusion criteria were articles published in a language other than English, review articles, case reports, grey literature, editorial comments, conference abstracts and animal studies. Studies involving special populations such as pregnant women or children were also excluded.

### Data extraction

Data extraction was independently conducted by two authors (WM, PA). The variables extracted from each study included: 1) study characteristics, i.e., the name of the first author, year of publication, ethnicity of the included population and study design; 2) patient characteristics, i.e., means and standard deviations (SD) of age, percentage of males, mean and SD of body mass index (BMI), percentage of AVS, mean and SD of duration of hypertension, mean and SD of number of anti-hypertensive medications used, mean and SD of defined daily dose (DDD) and mean and SD of duration of follow-up; and 3) criteria of clinical success employed in each study; 4) number of patients with complete clinical success versus partial or no clinical success after unilateral adrenalectomy was extracted where available. If that data was not available, the number of patients with complete plus partial clinical success versus no clinical success was extracted and 5) predictive factors of clinical success after unilateral adrenalectomy, including both clinical and laboratory predictors. Adjusted odds ratio, unadjusted odds ratio or crude data for the predictive factors were also collected.

### Data synthesis

Meta-analysis was performed using the STATA program version 16.0. (StataCorp LLC, College Station, TX, USA). For primary analysis, the adjusted odds ratio of predictive factors of clinical success reported in each study were used to calculate the pooled adjusted OR using a random effects model. For secondary analysis, pooled standardized mean differences (SMD) were calculated for crude continuous data and pooled OR for crude binary data. The predictive factors included in the meta-analysis should have been reported in at least two of the studies. Pooled OR were calculated using the logarithm of effect size and standard error from each study. Random effect modelling by the DerSimonian-Liard method was performed as the observed estimates of effect size can vary across studies due to sampling variability. The statistical significance level for this meta-analysis was set at p<0.05. To evaluate the statistical heterogeneity among the studies, the I^2^ statistic was assessed. I^2^ values >75% with a significant Cochran Q test (p<0.05) were considered to indicate high heterogeneity. I^2^ values of <75% were considered as moderate to high heterogeneity. Publication bias was assessed using funnel plots and Egger’s linear regression tests. Funnel plots should be a symmetrical inverted funnel when there is an absence of publication bias and asymmetrical when there is publication bias. A p-value of <0.05 was considered to indicate statistically significant publication bias for Egger’s regressions. For the predictors which contained publication bias, the effect size and 95% CI by the trim-and-fill method were also used to eliminate publication bias

As there was a difference in clinical success rate among studies which were AVS-guided and those that were non-AVS-guided (7), further subgroup analysis by AVS-guided adrenalectomy was also conducted to determine the effect of potential confounders. The subgroups were studies where all patients had performed AVS before adrenalectomy and studies with only some or none of the patients had undergone AVS before adrenalectomy. The included predictors for subgroup analysis should be reported in at least one study per each subgroup.

### Risk of bias assessment

The Newcastle-Ottawa scale (NOS) for cohort studies was used to assess risk of bias. The assessment was conducted by two authors independently (WM and PA) and discrepancies were resolved through discussion with the third author (PI). The NOS scale evaluated 3 domains: selection of study groups (4 points), comparability of groups (2 points), and outcomes (3 points) (17). Risk of bias is rated as low if NOS ≥7, moderate if NOS 4-6 and high if NOS ≤3.

## Results

**Figure 1:**
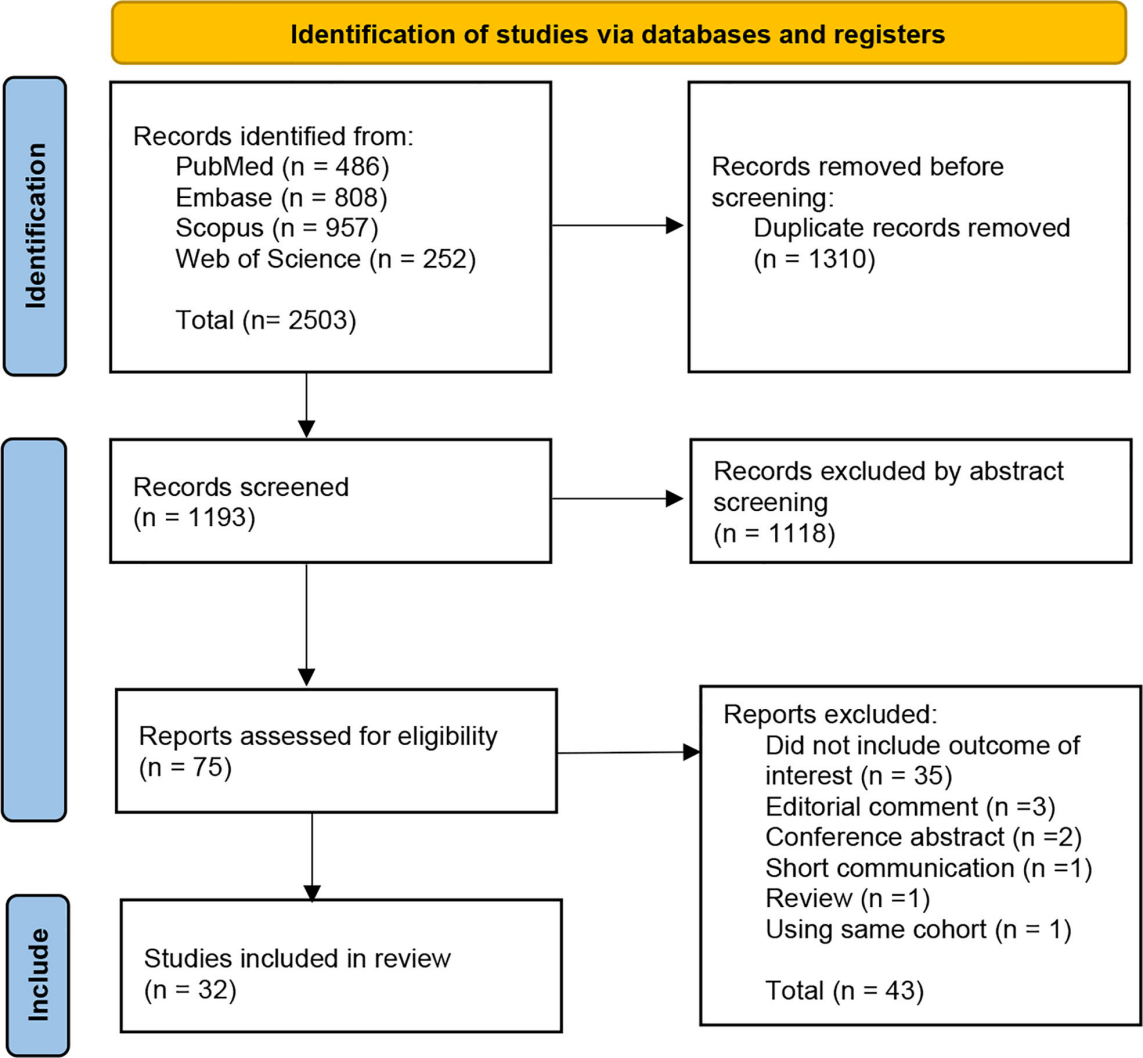
Prisma flow diagram.

A total of 2,503 articles were retrieved from database searches, including 486 from *PubMed*, 808 from *Embase*, 957 from *Scopus*, and 252 from *Web of Science.* From the retrieved articles, 1,310 duplicates were removed. A screening of titles and abstracts of the remaining 1,193 articles was performed which resulted in the exclusion of 1,118 additional articles which were not relevant to the objectives of this study. The full texts of the remaining 75 articles were retrieved and reviewed, resulting in the exclusion of an additional 43 articles due to a variety of reasons including consisting of editorial comments, conference abstracts, reviews or short communications; not providing outcomes of interest; and using the same cohort as other included studies. Finally, a total of 32 studies were included (9–12, 18–45). The PRISMA selection process used is shown in **Figure 1**.

### Study characteristics

**Table 1** shows the characteristics of the included studies. All 32 studies included were non-randomized cohort studies. Most of the studies (20 of 32) had been conducted in non-Asian populations. All studies provided data on age, sex and duration of follow-up. However, not all of the included studies provided data on BMI, percentage of AVS performed, number of anti-hypertensive medications, DDD and duration of hypertension. The majority (53%) of the clinical remission criteria used in the studies was Primary Aldosteronism Surgical Outcome (PASO) criteria. Other criteria used for determining clinical remission are as shown in **Table 1**. Thirteen of the 32 studies were multi-center, of which 3 were multi-continent international studies (9, 11, 12, 26, 31–34, 38, 39, 41, 43, 44). The clinical remission rates ranged from 15 to 82%. Eleven of the studies had performed AVS in all of the patients (9–12, 20, 29, 32, 38, 39, 42, 45). Four studies had compared complete and partial clinical success versus no clinical success (25, 33, 41, 45).

**Table 1:** Baseline characteristics of the 32 included studies.

Author	Year	Total patients	Complete clinical success (percentage)	Partial or absent clinical success (percentage)	Country	Study type	Mean age ± SD (year)	%Male	Mean BMI ± SD	AVS (%)	Mean number of anti-hypertensive medication ± SD	Mean DDD ± SD	Duration of hypertension± SD (years)	Remission criteria	Duration of follow-up (month)	NOS risk of bias
Pang (18)	2007	53	35 (66)	18 (34)	Australia	Cohort	50.2 ± 4.1	46.7	N/A	N/A	N/A	N/A	N/A	Other^1^	1-59	Moderate
Zarnegar (19)	2008	100	35 (35)	65 (65)	USA	Cohort	54.7 ± 12.1	50	28.9 ± 6.6	N/A	3 ± 1.3	N/A	10 ± 8.3	Other^2^	6	Low
Murashima (20)	2009	56	12 (21.4)	44 (78.6)	USA	Cohort	47.7 ± 6.4	58.9	N/A	100	2.5 ± 1.4	N/A	14.1 ± 10.4	Other^3^	9-34	Low
Kim (21)	2010	27	16 (59.2)	11 (40.8)	Korea	Cohort	45.3 ± 4	33.3	23.8 ± 3.1	N/A	2.3 ± 1.3	N/A	5.6 ± 7.8	Other^1^	6	Low
Linden (22)	2011	156	68 (43.5)	88 (56.5)	France	Cohort	45 ± 10.4	55	27.1 ± 4.7	55	1 ± 1.5	N/A	5 ± 6.7	Other^1^	1-6	Low
Wang (23)	2012	124	68 (54.8)	56 (45.2)	China	Cohort	48.8 ± 10.6	37.1	25.8 ± 6.3	N/A	2.1 ± 1	N/A	4.9 ± 2.5	Other^1^	6	Low
Zhang (24)	2013	376	207 (55)	169 (45)	China	Cohort	45.5 ± 10.6	63.6	24.1 ± 1.6	28.7	N/A	N/A	5.8 ± 2.1	Other^4^	6	Low
Hartmann (25)	2014	51	42 (82.3)	9 (17.7)	Czech Republic	Cohort	57 ± 9.5	43	30.7 ± 5.5	63	4.2 ± 1.3	N/A	N/A	Other^1^	12-600	Low
Wachtel (10)	2014	85	13 (15.3)	72 (84.7)	USA	Cohort	51.5 ± 10.9	62.2	31.7 ± 7.3	100	N/A	N/A	10 ± 11.9	Other^1^	6	Low
Utsumi (26)	2014	132	56 (42.4)	76 (57.6)	Japan^+^	Cohort	50.8 ± 11.9	55	22.9 ± 3.1	61.3	4 ± 2.9	N/A	9.3 ± 21.6	Other^4^	6	Low
Worth (27)	2015	58	13 (22.4)	45 (77.6)	USA	Cohort	52.6 ± 10.8	56.2	31.5 ± 7.3	74.1	N/A	N/A	13.5 ± 9.2	Other^5^	1-9	Low
Citton (28)	2015	122	55 (45)	67 (55)	Italy	Cohort	50.2 ± 11.6	50	26.1 ± 3.9	43.7	2.5 ± 1.2	N/A	8.8 ± 7.4	Other^1^	6-264	Low
Hannon (29)	2016	52	24 (46.1)	28 (53.9)	United Kingdom	Cohort	54 ± 13.2	57.7	N/A	100	3 ± 1.5	N/A	N/A	Other^1^	7-115	Low
Grytaas (30)	2017	52	11 (21.1)	41 (78.9)	Norway	Cohort	54 ± 13.8	37.9	N/A	88	3 ± 1.7	N/A	10 ± 7.5	Other^2^	24-192	Low
Williams (12)	2017	705	259 (36.7)	446 (63.3)	Multiple countries*	Cohort	50.8 ± 10.9	56	27.6 ± 5.2	100	N/A	3 ± 2.1	8 ± 8.5	PASO	6-12	Low
Umakoshi (11)	2018	377	95 (25.2)	282 (74.8)	Japan^+^	Cohort	52 ± 11.4	53.1	24.4 ± 4.2	100	N/A	2 ± 1.3	10.6 ± 9	PASO	6-12	Low
Sellgren (31)	2019	171	58 (33.9)	113 (66.1)	Sweden^+^	Cohort	52.6 ± 11	53	28.6 ± 5.5	82	N/A	3.7	N/A	PASO	1-24	Low
Morisaki (32)	2019	574	187 (32.6)	387 (67.4)	Japan^+^	Cohort	51.4 ± 11.7	49.3	24.1 ± 4.1	100	1.4 ± 3.7	N/A	8 ± 8.9	PASO	6	Low
Chan (33)	2019	236	188 (79.6)	48 (20.4)	Taiwan^+^	Cohort	49.8 ± 11	41.1	25.6 ± 4.3	N/A	N/A	N/A	7.4 ± 6.8	PASO	12	Low
Vorselaars (34)	2019	380	112 (29.4)	268 (70.5)	Multiple countries^#^	Cohort	50 ± 11	57	30 ± 6	64	3 ± 2.2	3.7 ± 2.6	8 ± 6.7	PASO	3-6	Low
Burrello (9)	2019	380	150 (39.5)	230 (60.5)	Multiple countries^α^	Cohort	50.5 ± 11.2	52.6	26.9 ± 5.2	100	N/A	2.5 ± 2.1	8.3 ± 8.4	PASO	6-12	Low
Bilige (35)	2019	126	58 (46)	68 (54)	China	Cohort	54.2 ± 12.2	41.2	26 ± 2.5	N/A	2.8 ± 1.1	N/A	5.1 ± 2.2	Other^1^	12-72	Low
Thiesmeyer (36)	2020	123	53 (43)	70 (57)	France, USA	Cohort	50.2 ± 10.6	57.6	N/A	36	2.6 ± 1.8	N/A	6.1 ± 8.4	PASO	2-20	Low
Picado (37)	2020	37	15 (40.5)	22 (59.5)	USA	Cohort	50 ± 10	41	30 ± 5	27	2 ± 1	N/A	10 ± 12.6	PASO	6	Low
Yang (38)	2020	150	97 (64.7)	53 (35.3)	China, Germany, Italy	Cohort	45.4 ± 12.3	34	23.4 ± 3.2	100	N/A	1.9	4.8 ± 6.6	PASO	6-12	Low
Saiki (39)	2020	322	116 (36)	206 (64)	Japan^+^	Cohort	54 ± 12.6	48.7	24.4 ± 4.1	100	1 ± 0.9	N/A	6 ± 8.1	PASO	6-12	Low
Wang (40)	2021	130	70 (53.8)	60 (46.2)	Japan	Cohort	44.9 ± 10.1	43.8	N/A	N/A	N/A	N/A	5.3 ± 5.7	PASO	6-24	Low
Chan (41)	2021	104	80 (76.9)	24 (23.1)	Singapore^+^	Cohort	50.8 ± 9.9	59.7	25.7 ± 3.7	43.26	N/A	2.2 ± 1.8	10.1 ± 8.2	PASO	6-12	Low
Dominguez (42)	2021	102	83 (81.3)	19 (18.7)	USA	Cohort	50 ± 10.4	54.9	31 ± 6.7	100	3 ± 0.9	N/A	10 ± 11.1	PASO	6-12	Low
Romero-Velez (43)	2021	53	22 (41.5)	31 (58.5)	USA, Mexico	Cohort	44 ± 13	45.3	28 ± 5	41.5	N/A	N/A	4.2 ± 4.6	PASO	9-12	Low
Leung (44)	2021	103	50 (48.5)	53 (51.5)	Hongkong^+^	Cohort	49.6 ± 9.5	55.8	N/A	N/A	2 ± 1.5	N/A	5.5 ± 5.9	PASO	6-12	Low
Morup (45)	2022	84	62 (73.8)	22 (26.2)	Denmark	Cohort	52 ± 11.4	49.5	28.4 ± 5.8	100	2.2 ± 1.3	N/A	8.1 ± 6.0	PASO	6-12	Low

*Germany, Netherlands, Japan, France, Australia, USA, Italy, Poland, Slovenia/.

^#^Europe, Canada, Australia, USA.

^α^Germany, Australia, Netherlands, Italy, Japan, Poland.

^+^Multi-center study.

N/A, Not available; SD, Standard deviation; DDD, Defined daily dose; ARS, Aldosteronoma Resolution Score.

PASO, The Primary Aldosteronism Outcome; Complete clinical success – Normal blood pressure without the aid of antihypertensive medication; Partial clinical success – the same blood pressure as before surgery with less antihypertensive medication or a reduction in blood pressure with either the same amount or less of antihypertensive medication; Absent clinical success – Unchanged or increased blood pressure with either the same amount or an increase in antihypertensive medication

^1^Clinical cure – normotensive, SBP ≤140 and DBP ≤90 and no antihypertensive medications; Improved control – normotensive and equal or fewer anti-hypertensives postoperatively or hypertensive and requiring fewer antihypertensives; No difference or worse control – hypertensive with the same or more antihypertensives postoperatively.

^2^Clinical cure – no hypertension defined as SBP < 140 and DBP <90 and not taking any antihypertensive medications 6 months after surgery; Persistent hypertension – either of 2 criteria were met 6 months postoperatively: persistent hypertension, SBP >140 or DBP> 90, or continued need of antihypertensive medications to adequately control blood pressure.

^3^Clinical cure – BP <140/90 without antihypertensive medications; Improvement: SBP decreased by >10 mmHg with the same number of drugs or SBP remained within 10 mmHg of preoperative value with fewer antihypertensives; No change in the number of antihypertensives required to keep BP within 10 mmHg of pre-operative value or BP increased by >10 mmHg with fewer medications; Worsening– more medications required to control BP or BP increased with the same medications

^4^Complete clinical cure – normotension without taking any antihypertensive agents 6 months postoperatively; Improved – normotension but with the need of an equal number or fewer antihypertensive agents to control BP, or hypertension but requiring fewer antihypertensive agents to control; Refractory – continued hypertension with an equal number or additional antihypertensive agents 6 months postoperatively.

^5^Clinical cure– patients completely off all antihypertensives with normalized blood pressure <130 mm Hg of SBP; Improved – reduction in medications by ≥ 33% with mean postoperative systolic blood pressure ≤ 130 mm Hg; No improvement – Individuals not meeting either of these end points.

### Risk of bias in the studies

Risk of bias was assessed using NOS for cohort studies (**Table 1** **)**. Most of the studies (31 of 32) evidenced high quality with a low risk of bias (9–12, 19–45). One study had moderate quality with a moderate risk of bias (18) as the comparable cohort was not adjusted or matched for confounders and had an inadequately short cohort follow-up time. Details of the NOS of the included studies are shown in the **Supplementary Table**.

### Results of syntheses

A total of 32 studies comprising 5,601 patients were included in this meta-analysis. In the primary analysis, the pooled adjusted odds ratios of 13 predictive factors were determined. The median number of studies reporting these predictive factors with adjusted odds ratios was 6 (range 2–14). In terms of demographics, older patients had a lower clinical success rate after adrenalectomy than younger patients (OR 0.97; 95% CI 0.94–0.99; p=0.01; I^2^ 19.29, Q-test p-value 0.27). Females had a higher rate of clinical success than males (OR 2.81; 95% CI 2.06–3.83; p<0.001; I^2^ 39.65, Q-test p-value 0.007). Patients with higher BMI had a lower clinical success rate than those with a lower BMI (OR 0.86; 95% CI 0.76–0.98; p=0.02; I^2^ 78.78, Q-test p-value <0.001). Corrected OR by trim-and-fill method for BMI showed an OR of 0.94; 95% CI 0.81-1.09. In terms of hypertensive status, a longer duration of hypertension or a higher number of antihypertensive medications used was associated with a lower clinical success rate than a shorter duration of hypertension and lower number of medications used (OR 0.92; 95% CI 0.88–0.96; p<0.001; I^2^ 90.56, Q-test p-value <0.001 and OR 0.44; 95% CI 0.29–0.67; p<0.001; I^2^ 75.20, Q-test p-value <0.001, respectively). The corrected OR by the trim-and-fill method for duration of hypertension was 0.95; 95% CI 0.91-0.99 and for the number of antihypertensive medications was 0.51 (0.33,0.79). Other predictive factors, including DDD, diabetes mellitus, systolic blood pressure, preoperative aldosterone level, serum potassium, presence or absence of target organ damage, eGFR and tumor size, revealed no significant association with clinical success after unilateral adrenalectomy. The primary analysis of adjusted odds ratios are shown in **Table 2**. The forest plot of the predictors using fully adjusted odds ratios which were significantly associated with clinical success are as shown in **Supplementary Figure**.

**Table 2:** Meta-analysis of predictive factors of clinical success after adrenalectomy in primary aldosteronism using fully adjusted odds ratio.

Predictors	Number of studies	Heterogeneity		Pooled odds ratio (95% CI)	p-value by random effects model	Egger’s test p-value
		**I^2^ (%)**	**Q-test**			
Age	9	19.29	0.27	0.97 (0.94, 0.99)	0.01	0.780
Female	13	39.65	0.007	2.81 (2.06, 3.83)	<0.001	0.759
BMI	8	78.78	<0.001	0.86 (0.76, 0.98) 0.94 (0.81, 1.09)*	0.02	<0.001
Duration of hypertension	14	90.56	<0.001	0.92 (0.88, 0.96) 0.95 (0.91, 0.99)*	<0.001	<0.001
Number of antihypertensive medications	12	75.20	<0.001	0.44 (0.29, 0.67) 0.51 (0.33,0.79)*	<0.001	0.038
Defined daily dose	4	80.06	<0.001	0.85 (0.68, 1.07)	0.17	0.724
Diabetes mellitus	2	90.8	<0.001	1.14 (0.10, 12.48)	0.91	N/A
Systolic blood pressure	8	82.65	<0.001	1.01 (0.97, 1.04)	0.75	0.424
Preoperative aldosterone level	6	87.69	<0.001	1.00 (0.97. 1.04)	0.77	0.502
Serum potassium	2	0.0	0.96	0.69 (0.47, 1.01)	0.06	N/A
Absence of target organ damage	3	86.6	<0.001	1.10 (0.33, 3.71) 2.84 (0.87, 9.19)*	0.88	<0.001
eGFR	2	1.17	0.31	1.01 (0.98. 1.04)	0.48	N/A
Tumor size	2	47.22	0.17	1.14 (0.79, 1.66)	0.48	N/A

*Trim and fill method

For secondary analysis, crude data of the 19 predictive factors were employed. The median number of studies reporting these predictive factors with crude data was 17 (range 3–23). Among the demographic data, age, sex and BMI were significantly related to clinical success rate. Younger patients had a higher rate of clinical success after adrenalectomy than older patients with an SMD of 0.54 years (95% CI -0.66,-0.42; p<0.001; I^2^ 52.49, Q-test p-value <0.001). Females had a higher clinical success rate than males (OR 2.96; 95% CI 2.21–4.14; p<0.001; I^2^ 73.70, Q-test p-value <0.001). The corrected OR by the trim-and-fill method for females was 2.34; 95% CI 1.68- 3.27. Patients with lower BMI had a higher rate of clinical success with an SMD of 0.49 kg/m^2^ (95% CI -0.58,-0.39; p<0.001; I^2^ 16.78, Q-test p-value 0.26). Lower blood pressure was observed in patients with clinical success than those without clinical success including systolic blood pressure (SMD -0.37 mmHg (95% CI -0.56,-0.18; p<0.001; I^2^ 81.37, Q-test p-value <0.001) and diastolic blood pressure (SMD -0.19 mmHg (95% CI -0.33,-0.06; p<0.001; I^2^ 59.32, Q-test p-value <0.001). In terms of hypertensive status, shorter duration of hypertension and a lower number of antihypertensive medications used was found in the clinical success patients than the patients without clinical success with SMD of 0.72 years; 95% CI -0.97,-0.46; p<0.001; I^2^ 88.60, Q-test p-value <0.001 and SMD of -0.81 drugs; 95% CI -1.09,-0.54; p<0.001; I^2^ 85.67, Q-test p-value <0.001. Laboratory investigations found serum potassium and eGFR were associated with clinical success rate. In patients with clinical success, lower serum potassium and higher eGFR were observed than in patients without clinical success with SMD of -0.16 mEq/L; 95% CI -0.28,-0.04; p=0.01; I^2^ 42.27, Q-test p-value =0.03 and SMD of 0.51 mL/min/1.73m^2^; 95% CI 0.16,0.87; p<0.001; I^2^ 71.87, Q-test p-value =0.01, respectively. Other laboratory investigations, including preoperative aldosterone, renin and aldosterone/renin ratio, did not show an association with clinical success. For underlying diseases, the incidence of dyslipidemia and diabetes mellitus were significantly lower in those with clinical success than those without clinical success (OR 0.29; 95% CI 0.15–0.58; p<0.001; I^2^ 56.94, Q-test p-value=0.10 and OR 0.36; 95% CI 0.22–0.59; p<0.001; I^2^ 47.90, Q-test p-value=0.05, respectively). Corrected OR by the trim-and-fill method for diabetes mellitus was 0.56; 95%CI 0.34-0.91. Left ventricular hypertrophy did not demonstrate a link with clinical success. The forest plot of the predictors using crude data which were significantly associated with clinical success are shown in **Supplementary Figure**.

### Subgroup analysis

Subgroup analysis categorized by studies in which all patients had AVS and the studies in which some or an unknown percentage of patients had AVS was conducted. As shown in **Table 3**, the results of the pooled adjusted odds ratio for predictive factors of clinical success did not change significantly (p-value of group differences >0.05) except for DDD of antihypertensive medications (p-value of group differences=0.03). Significant association of DDD with clinical success was demonstrated only in the studies which performed AVS in some patients or where the AVS status was unknown, while in the studies which performed AVS in all patients DDD was not associated with clinical success. However, the results of heterogeneity for age, female and systolic blood pressure showed significant improvement after subgroup analysis by AVS.

**Table 3:** Meta-analysis of predictive factors of clinical success after adrenalectomy in primary aldosteronism using crude data.

Predictors	Number of studies	Heterogeneity		Effect selection	Effect size (95% CI)	p-value by random effects model	Egger’s test p-value
		I^2^ (%)	Q-test				
Age	22	52.49	<0.001	SMD	-0.54 (-0.66, -0.42)	<0.001	0.802
Female	23	73.70	<0.001	OR	2.96 (2.21, 4.14) 2.34 (1.68, 3.27)*	<0.001	0.027
BMI	17	16.78	0.26	SMD	-0.49 (-0.58, -0.39)	<0.001	0.578
Systolic blood pressure	19	81.37	<0.001	SMD	-0.37 (-0.56, -0.18)	<0.001	0.665
Diastolic blood pressure	17	59.32	<0.001	SMD	-0.19 (-0.33, -0.06)	<0.001	0.788
Duration of hypertension	19	88.60	<0.001	SMD	-0.72 (-0.97, -0.46)	<0.001	0.677
Number of antihypertensive medications	17	85.67	<0.001	SMD	-0.81 (-1.09, -0.54)	<0.001	0.294
Defined daily dose	3	91.86	<0.001	SMD	-0.25 (-0.84, 0.35) -0.79 (-1.41, 0.17)*	0.41	0.004
Family history of hypertension	12	22.06	0.23	OR	0.93 (0.73, 1.20)	0.58	0.065
Tumor size	11	57.79	0.01	SMD	0.00 (-0.20, 0.20)	0.99	0.646
Serum potassium	18	42.27	0.03	SMD	-0.16 (-0.28, -0.04)	0.01	0.806
eGFR	4	71.87	0.01	SMD	0.51 (0.16, 0.87)	<0.001	0.922
Preoperative aldosterone level	20	83.00	<0.001	SMD	-0.17 (-0.38, 0.05)	0.13	0.213
Preoperative plasma renin activity	17	17.41	0.25	SMD	-0.02 (-0.12, 0.08)	0.69	0.624
Aldosterone-renin ratio	15	79.72	<0.001	SMD	-0.02 (-0.24, 0.20) 0.16 (-0.06, 0.40)*	0.88	0.002
Preoperative adrenal venous sampling	9	52.06	0.03	OR	1.16 (0.70, 1.94)	0.56	0.687
Dyslipidemia	3	56.94	0.10	OR	0.29 (0.15, 0.58)	<0.001	0.503
Diabetes mellitus	9	47.90	0.05	OR	0.36 (0.22, 0.59) 0.56 (0.34, 0.91)*	<0.001	0.003
Left ventricular hypertrophy	4	75.63	0.01	OR	0.61 (0.30, 1.25)	0.17	0.650

*Trim and fill method.

The results of subgroup analysis by AVS of crude data for predictors of clinical success also did not show significant changes except for serum potassium (p-value of group differences=0.03). Significant association of serum potassium with clinical success was demonstrated only in the studies which performed AVS in some patients or in patients with unknown status, while in studies which performed AVS in all patients, serum potassium was not associated with clinical success. The results of heterogeneity for age, systolic blood pressure, diastolic blood pressure, tumor size, serum potassium, preoperative aldosterone level and left ventricular hypertrophy revealed significant improvement after subgroup analysis by AVS. Data are as shown in **Table 4**.

**Table 4:** Subgroup analysis of the predictive factors of clinical success after adrenalectomy in primary aldosteronism by adrenal venous sampling status using fully adjusted odds ratio .

Predictors	Number of studies	Heterogeneity		Pooled odds ratio (95% CI)	p-value of group differences
		I^2^ (%)	Q-test		
Age					
- AVS in some patients - AVS in all patients	4 5	47.95 0.00	0.12 0.76	0.98 (0.93, 1.04) 0.95 (0.93, 0.98)	0.28
Female					
- AVS in some patients - AVS in all patients	9 4	45.73 0.00	0.06 0.17	2.60 (1.69, 4.02) 0.95 (0.93, 0.98)	0.28
BMI					
- AVS in some patients - AVS in all patients	3 4	82.23 75.91	<0.001 <0.001	0.45 (0.18, 1.11) 0.93 (0.83, 1.04)	0.12
Duration of hypertension					
- AVS in some patients - AVS in all patients	8 6	89.15 91.98	<0.001 <0.001	0.74 (0.62, 0.88) 0.78 (0.67, 0.92)	0.61
Number of antihypertensive medications					
- AVS in some patients - AVS in all patients	8 4	78.67 73.82	<0.001 0.01	0.42 (0.23, 0.74) 0.46 (0.23, 0.94)	0.93
Defined daily dose					
- AVS in some patients - AVS in all patients	1 3	- 82.28	- 0	0.56 (0.38, 0.83) 0.92 (0.73, 1.17)	0.03
Systolic blood pressure					
- AVS in some patients - AVS in all patients	6 2	80.60 0.00	<0.001 0.99	1.01(0.95, 1.07) 0.99 (0.98, 1.00)	0.59
Preoperative aldosterone level					
- AVS in some patients - AVS in all patients	5 1	85.97 -	<0.001 -	0.99 (0.95, 1.04) 1.03 (1.02, 1.05)	0.11

### Publication bias

In the primary analysis of the adjusted odds ratio, Egger’s regression test revealed publication bias for BMI, duration of hypertension and number of antihypertensive medications used as well as absence of target organ damage. Among the crude data, predictive factors including being female, DDD, aldosterone-renin ratio and diabetes mellitus showed publication bias. Data are shown in **Tables 2** and **5**. Funnel plots and funnel plots with the trim-and-fill method for each of the predictive factors are provided in **Supplementary Figure**.

**Table 5:** Meta-analysis of predictive factors of clinical success after adrenalectomy in primary aldosteronism using crude data.

Predictors	Number of studies	Heterogeneity		Effect selection	Effect size (95% CI)	p-value by random effects model	Egger’s test p-value
		I^2^ (%)	Q-test		
Age	22	52.49	<0.001	SMD	-0.54 (-0.66, -0.42)	<0.001	0.802
Female	23	73.70	<0.001	OR	2.96 (2.21, 4.14) 2.34 (1.68, 3.27)*	<0.001	0.027
BMI	17	16.78	0.26	SMD	-0.49 (-0.58, -0.39)	<0.001	0.578
Systolic blood pressure	19	81.37	<0.001	SMD	-0.37 (-0.56, -0.18)	<0.001	0.665
Diastolic blood pressure	17	59.32	<0.001	SMD	-0.19 (-0.33, -0.06)	<0.001	0.788
Duration of hypertension	19	88.60	<0.001	SMD	-0.72 (-0.97, -0.46)	<0.001	0.677
Number of antihypertensive medications	17	85.67	<0.001	SMD	-0.81 (-1.09, -0.54)	<0.001	0.294
Defined daily dose	3	91.86	<0.001	SMD	-0.25 (-0.84, 0.35) -0.79 (-1.41, 0.17)*	0.41	0.004
Family history of hypertension	12	22.06	0.23	OR	0.93 (0.73, 1.20)	0.58	0.065
Tumor size	11	57.79	0.01	SMD	0.00 (-0.20, 0.20)	0.99	0.646
Serum potassium	18	42.27	0.03	SMD	-0.16 (-0.28, -0.04)	0.01	0.806
eGFR	4	71.87	0.01	SMD	0.51 (0.16, 0.87)	<0.001	0.922
Preoperative aldosterone level	20	83.00	<0.001	SMD	-0.17 (-0.38, 0.05)	0.13	0.213
Preoperative plasma renin activity	17	17.41	0.25	SMD	-0.02 (-0.12, 0.08)	0.69	0.624
Aldosterone-renin ratio	15	79.72	<0.001	SMD	-0.02 (-0.24, 0.20) 0.16 (-0.06, 0.40)*	0.88	0.002
Preoperative adrenal venous sampling	9	52.06	0.03	OR	1.16 (0.70, 1.94)	0.56	0.687
Dyslipidemia	3	56.94	0.10	OR	0.29 (0.15, 0.58)	<0.001	0.503
Diabetes mellitus	9	47.90	0.05	OR	0.36 (0.22, 0.59) 0.56 (0.34, 0.91)*	<0.001	0.003
Left ventricular hypertrophy	4	75.63	0.01	OR	0.61 (0.30, 1.25)	0.17	0.650

*Trim and fill method.

## Discussion

This systematic review and meta-analysis is the first to describe and quantify the degree of predictive factors associated with clinical success after unilateral adrenalectomy in patients with PA. The present study found that multiple predictive factors are associated with higher rates of clinical success after unilateral adrenalectomy. Based on fully adjusted odds ratios, younger age, being female, a lower BMI, shorter duration of hypertension and lower number of medications used were significantly related to the clinical success rate. Further analysis of the predictors using crude data demonstrated that lower systolic and diastolic blood pressure, lower serum potassium, higher eGFR and the absence of diabetes and dyslipidemia were also associated with a higher incidence of clinical success after adrenalectomy.

Interestingly, younger patients and female patients had better chance of clinical success than older patients and male patients. A previous study showed that mildly decreased renin and aldosterone levels were observed in elderly patients (46), so the levels of aldosterone and renin may not explain this association. A high postoperative incidence of persistent hypertension and hyperkalemia were observed in elderly patients, especially in individuals with long-standing hypertension (47). A probable explanation is that in the elderly essential hypertension is more prevalent than in younger patients, thus leading to persistent hypertension even after unilateral adrenalectomy (48). However, the reason for the association between age and decreased success rate is still unclear and needs further study. The underlying mechanism behind the association between female sex and higher success rates remains unclear. Previous human and animal studies have shown that estrogen may have a protective effect on salt-sensitive hypertension and may possibly also have a vasoprotective effect by suppressing renin-angiotensin-aldosterone system activity (49, 50). Lower BMI tended to be associated with clinical success after adrenalectomy. The physiological factors behind this finding are uncertain. This relationship cannot be explained by a high level of aldosterone as a study showed that BMI has a positive correlation with plasma aldosterone levels in essential hypertension patients but not in PA patients (51). As with older patients, obese patients may have a higher prevalence of essential hypertension, a component of metabolic syndrome which can cause persistent hypertension after definite treatment of PA. A shorter duration of hypertension and a lower number of antihypertensive medications used also predicts a higher success rate. Patients with long-standing hypertension from chronic exposure to aldosterone and who required multiple medications showed an increased chance of vascular remodelling involving increased intima-media thickness and arterial stiffness which can lead to a higher risk of cardiovascular disease, chronic kidney disease and metabolic syndrome (52). These changes in vascular morphology may indicate a lower chance of success even after specific treatment of PA.

From the analysis based on crude data, lower blood pressure, which indicates a lower severity of disease, was significantly associated with a higher chance of success. Vascular remodelling may play a role in this association. A study revealed that in severe hypertension, there is less of a compensatory mechanism to counteract elevated blood pressure, leading subsequently to vascular damage (53). Moreover, higher eGFR, absence of diabetes and dyslipidemia are associated with a higher rate of clinical success. Again, the underpinning explanation of that association could be that increased and irreversible vascular damage can be observed in patients with chronic kidney disease, dyslipidemia and diabetes over and above that caused by hypertension (54, 55). Thus, the chance of curing the hypertension is low even after the primary cause of hypertension has been eliminated by adrenalectomy in PA. An interesting and unexpected result which needs further clarification was that the lower the potassium level, the higher the rate of clinical success as a higher degree of hypokalemia means a greater severity of hyperaldosteronism (56), the predictors which were acquired from crude data should be interpreted with caution. The association between these predictive factors and clinical success does not represent a causal relationship since it is based on unadjusted data which could be confounded by multiple interfering factors.

AVS can interfere with the success of adrenalectomy. Higher rates of success were observed in AVS patients (7). Further subgroup meta-analysis categorized by AVS-guided and non-AVS-guided adrenalectomy has also been conducted. The majority of those results remained the same after subgroup analysis was performed. DDD of medications and serum potassium showed different results after subgroup analysis. However, the DDD results should be interpreted with caution as there was only one study in a subgroup of patients who had AVS. After subgroup analysis, the majority of the predictors showed improvement in terms of heterogeneity. It could be implied that performing AVS modified the effect of clinical success for most of the predictors.

One of the strengths of this first meta-analysis is that only studies which had pre-defined and clear criteria for clinical success were included. Also, funnel plots and Egger’s test indicated that there was publication bias in some of the predictive factors which indicates that many negative unpublished results were not published. Funnel plots with the trim-and-fill method were also applied in this meta-analysis to rectify the corrected effect size. Most of the results remained constant after the trim-and-fill method with the exception of BMI with fully adjusted OR which showed a non-significant association following trim-and-fill. Nevertheless, the results of the association with BMI from crude data remained the same after the trim-and-fill method was applied. Another strength of this study is that the subgroup analysis of whether the included studies had conducted AVS before adrenalectomy was performed or not to reduce the effect modification from AVS. The information acquired from this meta-analysis can be utilized in multiple ways. These predictors can help clinicians identify patients who may have a lower chance of clinical success and so begin early monitoring, early re-initiation of antihypertensive medications and close follow-up after adrenalectomy. Conversely, in patients with multiple predictors of success, clinicians may have higher confidence to proceed to adrenalectomy. In addition, these predictors can help clinicians provide advice to patients regarding the chance of cure and assist them in making a decision regarding adrenalectomy.

There are some limitations in this meta-analysis. First, multiple criteria of clinical success were employed by the different studies. The variety of criteria of clinical success may have affected the outcomes of this meta-analysis. PASO criteria of clinical cure of PA were first developed in 2017 and have been used mostly in the studies published after 2017. Second, the duration of follow-up among the studies ranged widely, from 1 month to 5 years. As some patients’ hypertensive status may have improved slowly after 5 years of adrenalectomy, this could have affected the outcomes especially clinical success rate of the meta-analysis. Third, there was a high level of heterogeneity among the studies. However, the subgroup analysis by AVS status showed significant improvement in terms of heterogeneity for the majority of the predictors. Lastly, the pooled adjusted odds ratio of the predictive factors in most of the included studies were presented as categorical data with different cut-off levels among the studies which may have affected the meta-analysis outcomes.

In summary, demographic data and laboratory investigations can help predict the likelihood of clinical success after unilateral adrenalectomy in PA patients. The success rate is higher in PA patients who are younger, female, have a lower BMI, a shorter duration of hypertension, use a lower number of medications, have lower systolic and diastolic blood pressure, lower serum potassium, higher eGFR and no history of diabetes or dyslipidemia. These predictors can be used in future research to develop numerical scores which could facilitate the prediction of clinical success after surgery and further improve the quality of care of PA patients.

## Data availability statement

The raw data supporting the conclusions of this article will be made available by the authors, without undue reservation.

## Author contributions

WM designed the study, collected, analyzed, and interpreted the data, and was the major contributor in writing the manuscript. PA collected and performed data analysis. PP performed the data analyses and edited the manuscript. PI performed data analysis, reviewed and edited the manuscript. All authors contributed to the article and approved the submitted version.

## Acknowledgments

The authors are grateful to Dr.Lamar G.Robert and Dr.Chongchit A.Robert for reviewing the manuscript.

## Conflict of interest

The authors declare that the research was conducted in the absence of any commercial or financial relationships that could be construed as a potential conflict of interest.

## Publisher’s note

All claims expressed in this article are solely those of the authors and do not necessarily represent those of their affiliated organizations, or those of the publisher, the editors and the reviewers. Any product that may be evaluated in this article, or claim that may be made by its manufacturer, is not guaranteed or endorsed by the publisher.
