# Supplementary material for: Predictive factors of clinical success after adrenalectomy in primary aldosteronism: A systematic review and meta-analysis
Source: Front Endocrinol (Lausanne). 2022 Aug 18;13:925591. doi: 10.3389/fendo.2022.925591 (PMC9434311; doi:10.3389/fendo.2022.925591)
Supplement: Supplementary file 1 [file DataSheet_1.docx]

**Supplementary appendix**

**Table S1.** Keywords for article searches

**Table S2.** Risk of bias assessment by Newcastle-Ottawa scale

**Figure S1.** Forest plot of the predictive factors significantly associated with complete clinical success after adrenalectomy using fully adjusted odds ratio

**Figure S2.** Forest plot of the predictive factors significantly associated with complete clinical success after adrenalectomy using crude data

**Figure S3.** Funnel plot of predictive factors using fully adjusted odds ratio

**Figure S4.** Funnel plot of predictive factors using crude data

**Figure S5.** Funnel plot with trim-and-fill method of predictive factors using fully adjusted odds ratio

**Figure S6.** Funnel plot with trim-and-fill method of predictive factors using crude data

**Table S1.** Keywords for article searches

| Keywords | Number of articles |
| --- | --- |
| *Pubmed* | 485 |
| ("Hyperaldosteronism"[Mesh] OR "primary aldosteronism" OR "primary hyperaldosteronism" OR "aldosteronism") AND ("Adrenalectomy"[Mesh] OR "surgical" OR "surgery" OR "unilateral adrenalectomy") AND ("Patient Outcome Assessment"[Mesh] OR "clinical outcome" OR "outcome" OR "predictor" OR "predictive factor") |  |
| *Embase* | 808 |
| ('primary hyperaldosteronism'/exp OR 'primary aldosteronism' OR 'primary hyperaldosteronism' OR 'aldosteronism') AND ('adrenalectomy'/exp OR 'surgical' OR 'surgery' OR 'unilateral adrenalectomy' OR 'adrenalectomy') AND ('outcome'/exp OR 'predictive factor'/exp OR 'clinical outcome' OR 'outcome' OR 'predictor' OR 'predictive factor') |  |
| *Scopus* | 957 |
| ( ( "Hyperaldosteronism"  OR  "primary aldosteronism"  OR  "primary hyperaldosteronism"  OR  "aldosteronism" )  AND  ( "Adrenalectomy"  OR  "surgical"  OR  "surgery"  OR  "unilateral adrenalectomy" )  AND  ( "Patient Outcome Assessment"  OR  "clinical outcome"  OR  "outcome"  OR  "predictor"  OR  "predictive factor" ) ) |  |
| *Web of Science* | 252 |
| ( ( "Hyperaldosteronism"  OR  "primary aldosteronism"  OR  "primary hyperaldosteronism"  OR  "aldosteronism" )  AND  ( "Adrenalectomy"  OR  "surgical"  OR  "surgery"  OR  "unilateral adrenalectomy" )  AND  ( "Patient Outcome Assessment"  OR  "clinical outcome"  OR  "outcome"  OR  "predictor"  OR  "predictive factor" ) ) |  |

**Table S2.** Risk of bias assessment by Newcastle-Ottawa scale

| **Study** | **Selection** | | | | **Comparability** | **Outcome** | | | **Total** |
| --- | --- | --- | --- | --- | --- | --- | --- | --- | --- |
|  | **Representativeness of the exposed cohort** | **Selection of the non-exposed cohort** | **Ascertainment of exposure** | **Demonstration that outcome of interest was not present at start of study** | **Comparability of cohorts on the basis of the design or analysis** | **Assessment of outcome** | **Was follow-up long enough for outcomes to occur** | **Adequacy of follow up of cohorts** |  |
| Pang 2007 | **🟑** | **🟑** | **🟑** | **🟑** | - | **🟑** | **🟑** | - | 6 |
| Zarnegar 2008 | **🟑** | **🟑** | **🟑** | **🟑** | **🟑** | **🟑** | **🟑** | **🟑** | 8 |
| Murashima 2009 | **🟑** | **🟑** | **🟑** | **🟑** | - | **🟑** | **🟑** | **🟑** | 7 |
| Kim 2010 | **🟑** | **🟑** | **🟑** | **🟑** | **🟑** | **🟑** | **🟑** | **🟑** | 8 |
| Linden 2011 | **🟑** | **🟑** | **🟑** | **🟑** | **🟑** | **🟑** | **🟑** | - | 7 |
| Wang 2012 | **🟑** | **🟑** | **🟑** | **🟑** | **🟑** | **🟑** | **🟑** | **🟑** | 8 |
| Zhang 2013 | **🟑** | **🟑** | **🟑** | **🟑** | **🟑** | **🟑** | **🟑** | **🟑** | 8 |
| Hartmann 2014 | **🟑** | **🟑** | **🟑** | **🟑** | - | **🟑** | **🟑** | **🟑** | 7 |
| Wachtel 2014 | **🟑** | **🟑** | **🟑** | **🟑** | **🟑** | **🟑** | **🟑** | **🟑** | 8 |
| Utsumi 2014 | **🟑** | **🟑** | **🟑** | **🟑** | **🟑** | **🟑** | **🟑** | **🟑** | 8 |
| Worth 2015 | **🟑** | **🟑** | **🟑** | **🟑** | - | **🟑** | **🟑** | **🟑** | 7 |
| Citton 2015 | **🟑** | **🟑** | **🟑** | **🟑** | - | **🟑** | **🟑** | **🟑** | 7 |
| Hannon 2016 | **🟑** | **🟑** | **🟑** | **🟑** | - | **🟑** | **🟑** | **🟑** | 7 |
| Grytaas 2017 | **🟑** | **🟑** | **🟑** | **🟑** | - | **🟑** | **🟑** | **🟑** | 7 |
| Williams 2017 | **🟑** | **🟑** | **🟑** | **🟑** | **🟑🟑** | **🟑** | **🟑** | **🟑** | 9 |
| Umakoshi 2018 | **🟑** | **🟑** | **🟑** | **🟑** | **🟑🟑** | **🟑** | **🟑** | **🟑** | 9 |
| Sellgren 2019 | **🟑** | **🟑** | **🟑** | **🟑** | - | **🟑** | **🟑** | **🟑** | 7 |
| Morisaki 2019 | **🟑** | **🟑** | **🟑** | **🟑** | - | **🟑** | **🟑** | **🟑** | 7 |
| Chan 2019 | **🟑** | **🟑** | **🟑** | **🟑** | - | **🟑** | **🟑** | **🟑** | 7 |
| Vorselaars 2019 | **🟑** | **🟑** | **🟑** | **🟑** | - | **🟑** | **🟑** | **🟑** | 7 |
| Burrello 2019 | **🟑** | **🟑** | **🟑** | **🟑** | **🟑** | **🟑** | **🟑** | **🟑** | 8 |
| Bilige 2019 | **🟑** | **🟑** | **🟑** | **🟑** | **🟑** | **🟑** | **🟑** | **🟑** | 8 |
| Thiesmeyer 2020 | **🟑** | **🟑** | **🟑** | **🟑** | - | **🟑** | **🟑** | **🟑** | 7 |
| Picado 2020 | **🟑** | **🟑** | **🟑** | **🟑** | **🟑** | **🟑** | **🟑** | **🟑** | 8 |
| Yang 2020 | **🟑** | **🟑** | **🟑** | **🟑** | **🟑** | **🟑** | **🟑** | **🟑** | 8 |
| Saiki 2020 | **🟑** | **🟑** | **🟑** | **🟑** | **🟑** | **🟑** | **🟑** | **🟑** | 8 |
| Wang 2021 | **🟑** | **🟑** | **🟑** | **🟑** | **🟑** | **🟑** | **🟑** | **🟑** | 8 |
| Chan 2021 | **🟑** | **🟑** | **🟑** | **🟑** | - | **🟑** | **🟑** | **🟑** | 8 |
| Dominguez 2021 | **🟑** | **🟑** | **🟑** | **🟑** | **🟑** | **🟑** | **🟑** | **🟑** | 8 |
| Romero-Velez 2021 | **🟑** | **🟑** | **🟑** | **🟑** | **🟑** | **🟑** | **🟑** | **🟑** | 8 |
| Leung 2021 | **🟑** | **🟑** | **🟑** | **🟑** | **🟑** | **🟑** | **🟑** | **🟑** | 8 |
| Morup 2022 | **🟑** | **🟑** | **🟑** | **🟑** | **🟑** | **🟑** | **🟑** | **🟑** | 8 |


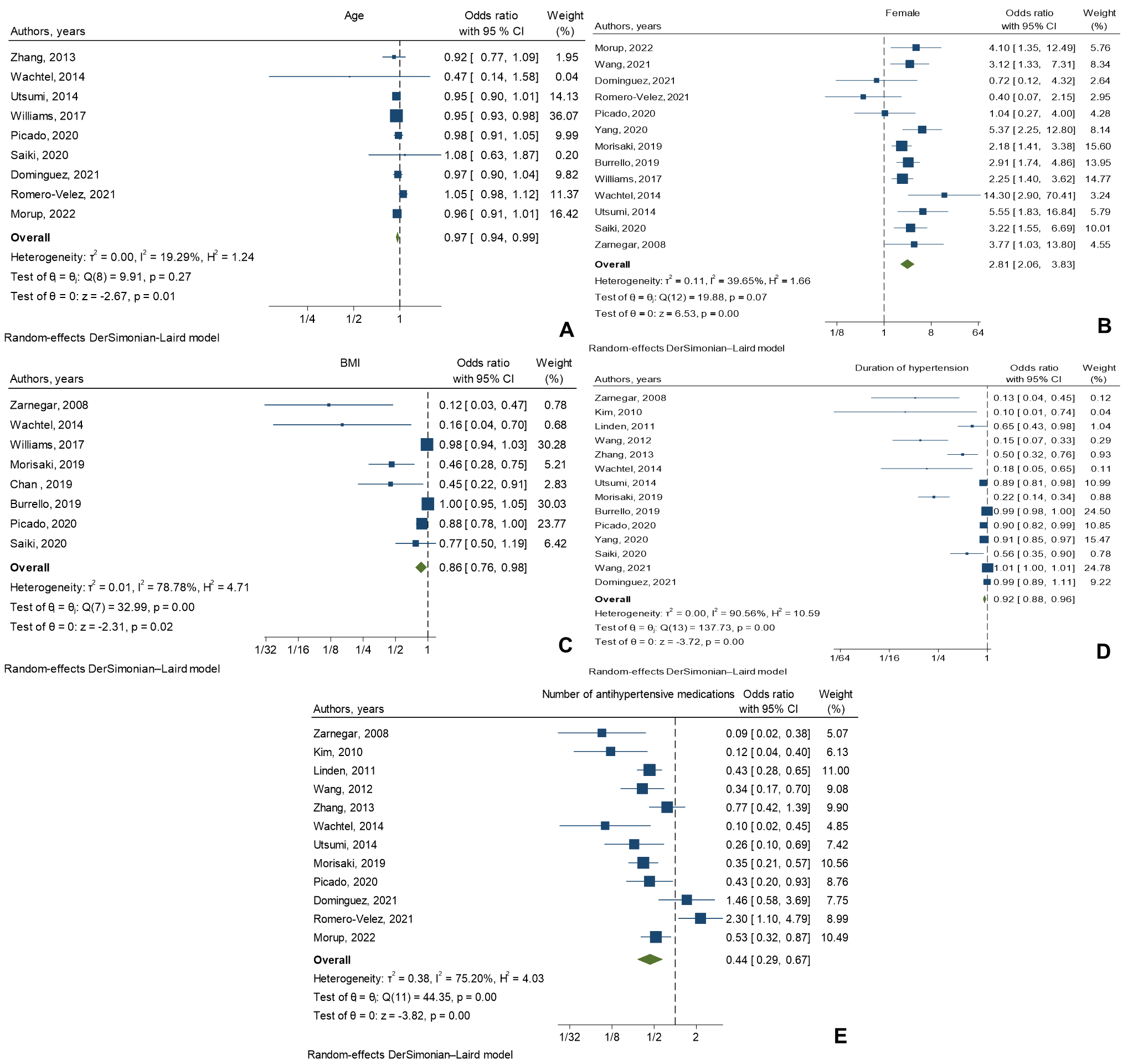


**Figure S1.** Forest plot of the predictive factors significantly associated with complete clinical success after adrenalectomy using fully adjusted odds ratio: age (A), female (B), BMI (C), duration of hypertension (D), and number of antihypertensive medications (E)

**
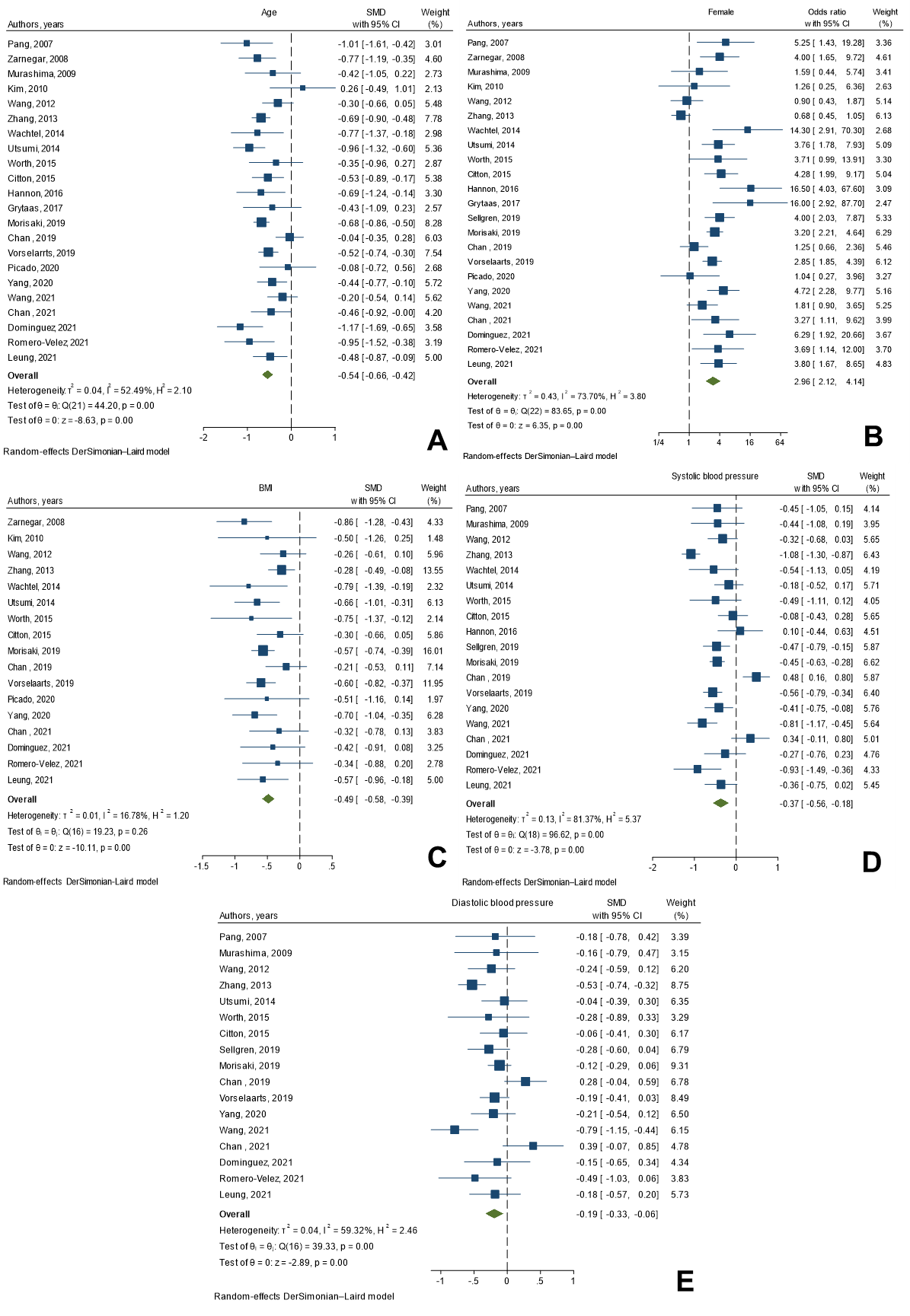
**

**Figure S2.** Forest plot of the predictive factors significantly associated with complete clinical success after adrenalectomy using crude data: age (A), female (B), BMI (C), systolic blood pressure (D), and diastolic blood pressure (E)

**
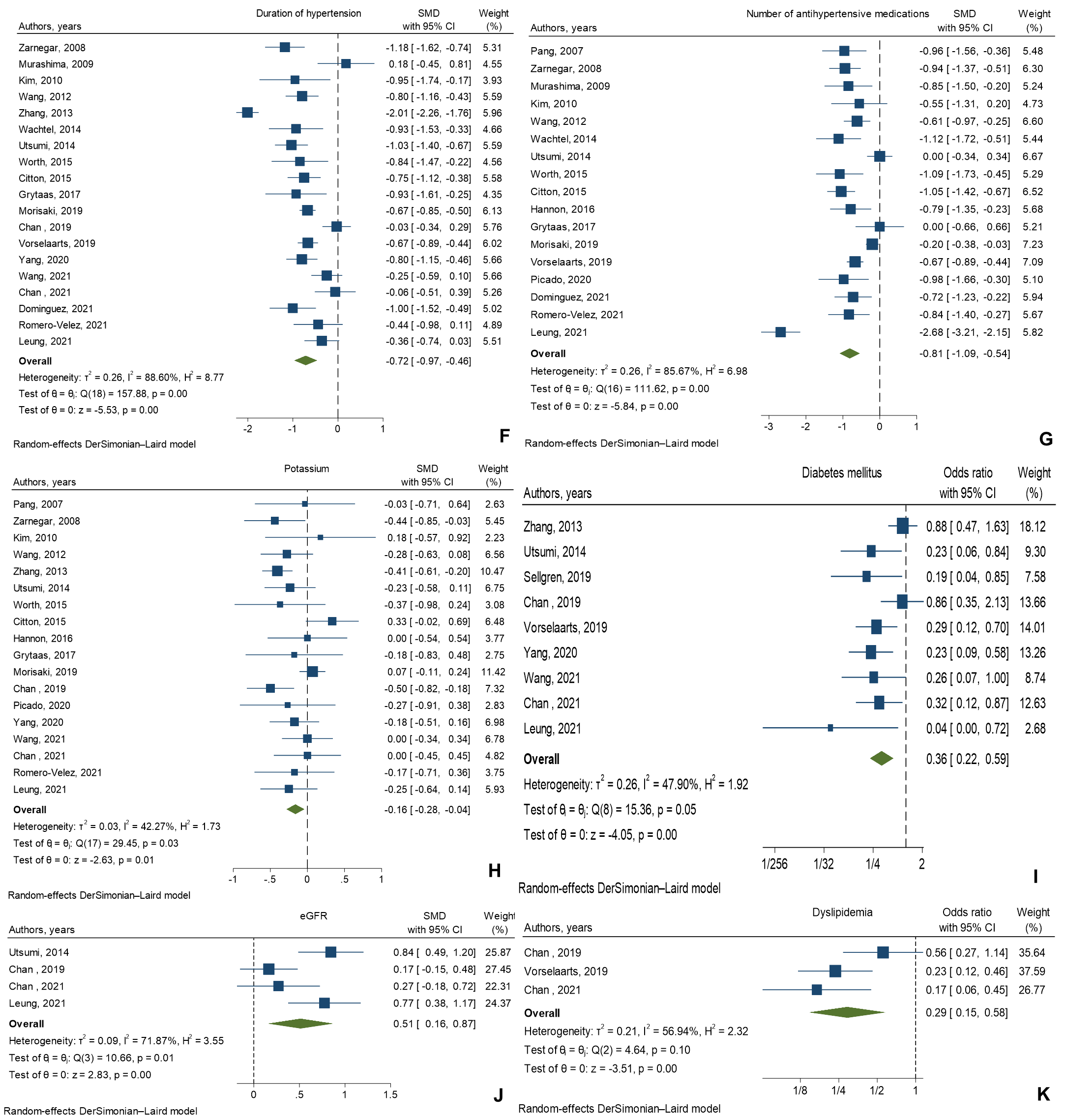
**

**Figure S2 (continued).** Forest plot of the predictive factors significantly associated with complete clinical success after adrenalectomy using crude data: duration of hypertension (F), number of antihypertensive medications (G), potassium (H), diabetes mellitus (I), eGFR (J), and (K) dyslipidemia

**
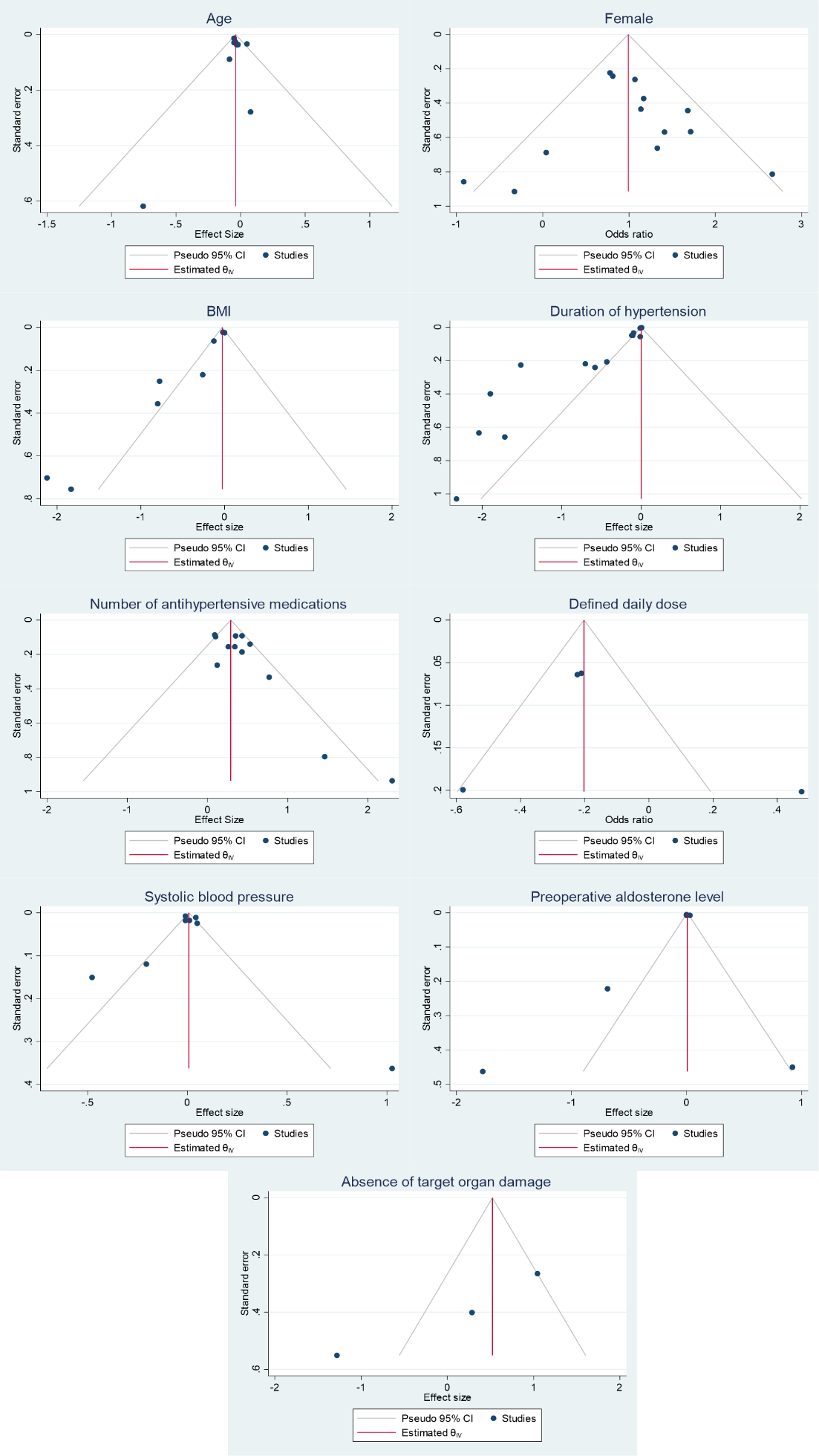
**

**Figure S3.** Funnel plot of predictive factors using fully adjusted odds ratio


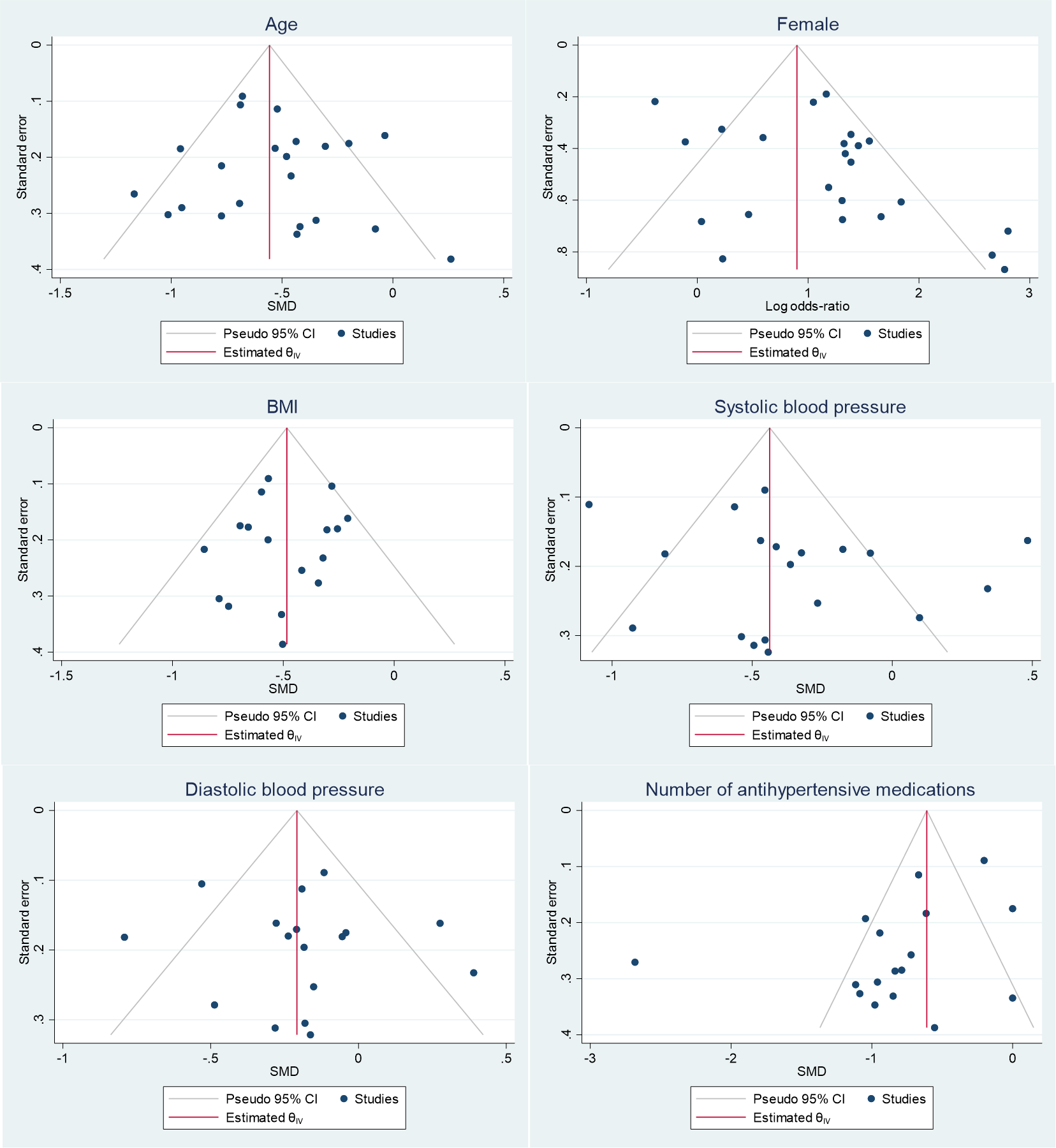


**Figure S4.** Funnel plot of predictive factors using crude data

**
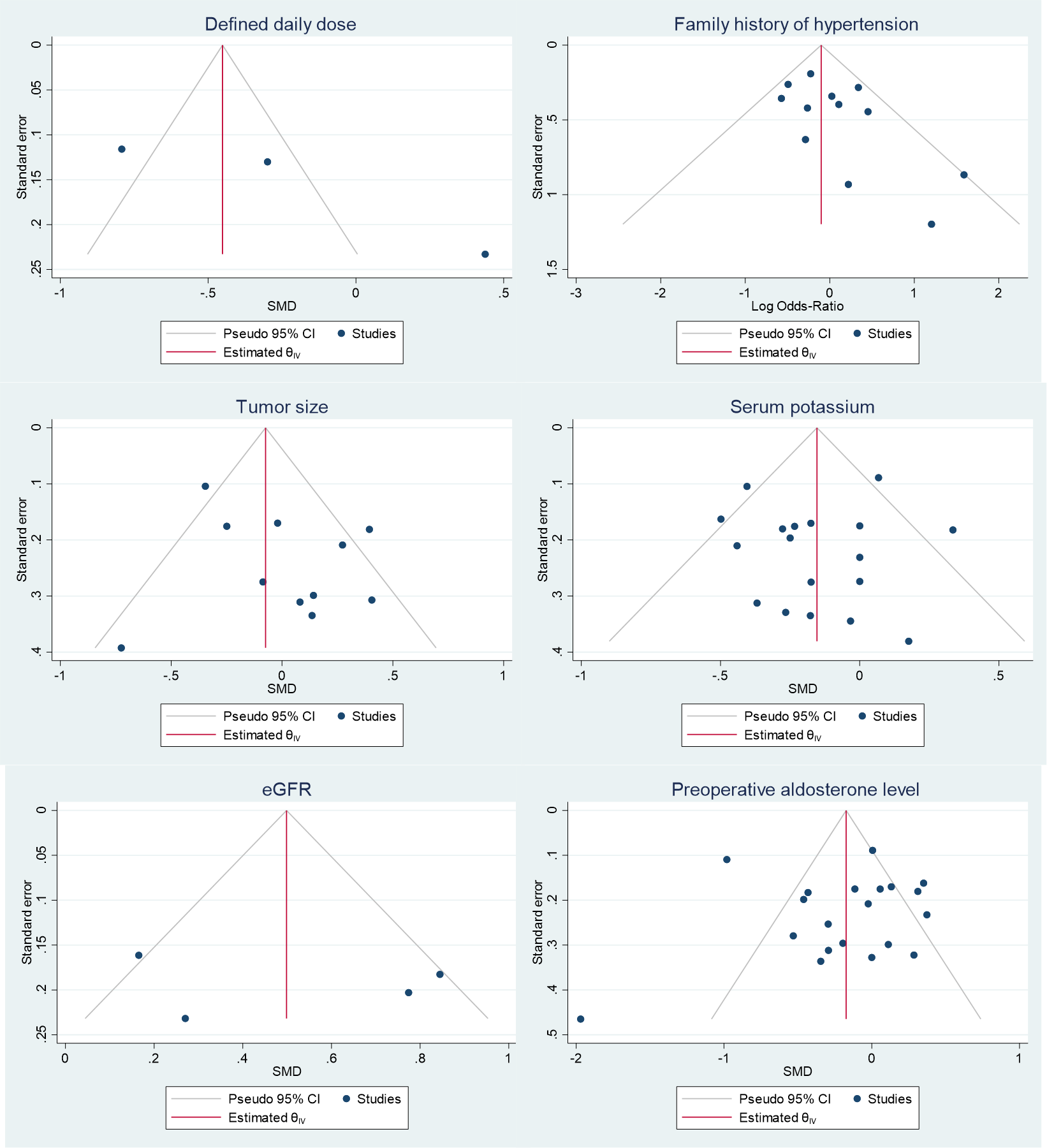
**

**Figure S4 (continued).** Funnel plot of predictive factors using crude data

**
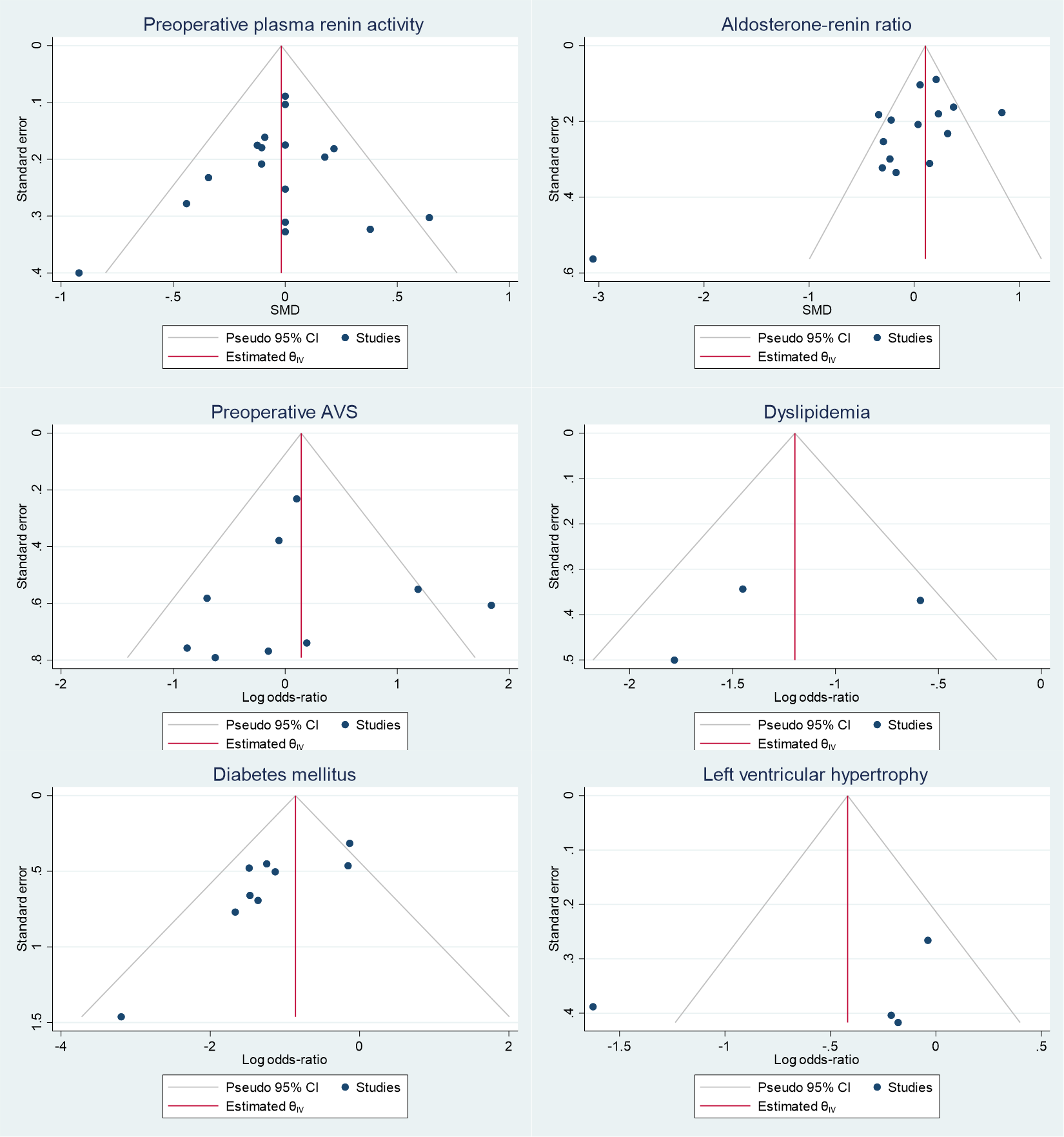
**

**Figure S4 (continued).** Funnel plot of predictive factors using crude data

**
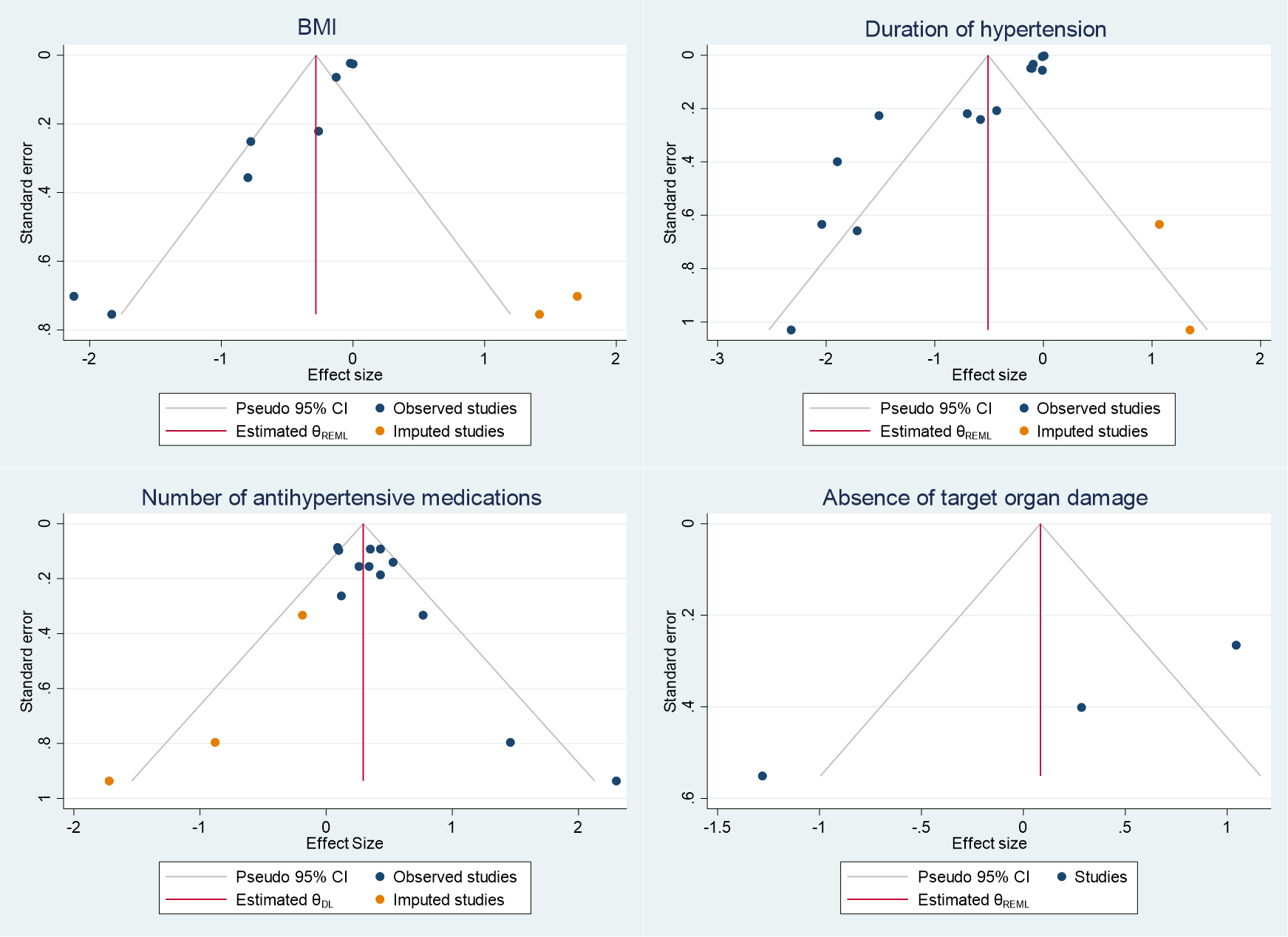
**

**Figure S5.** Funnel plot with trim-and-fill method of predictive factors using fully adjusted odds ratio

**
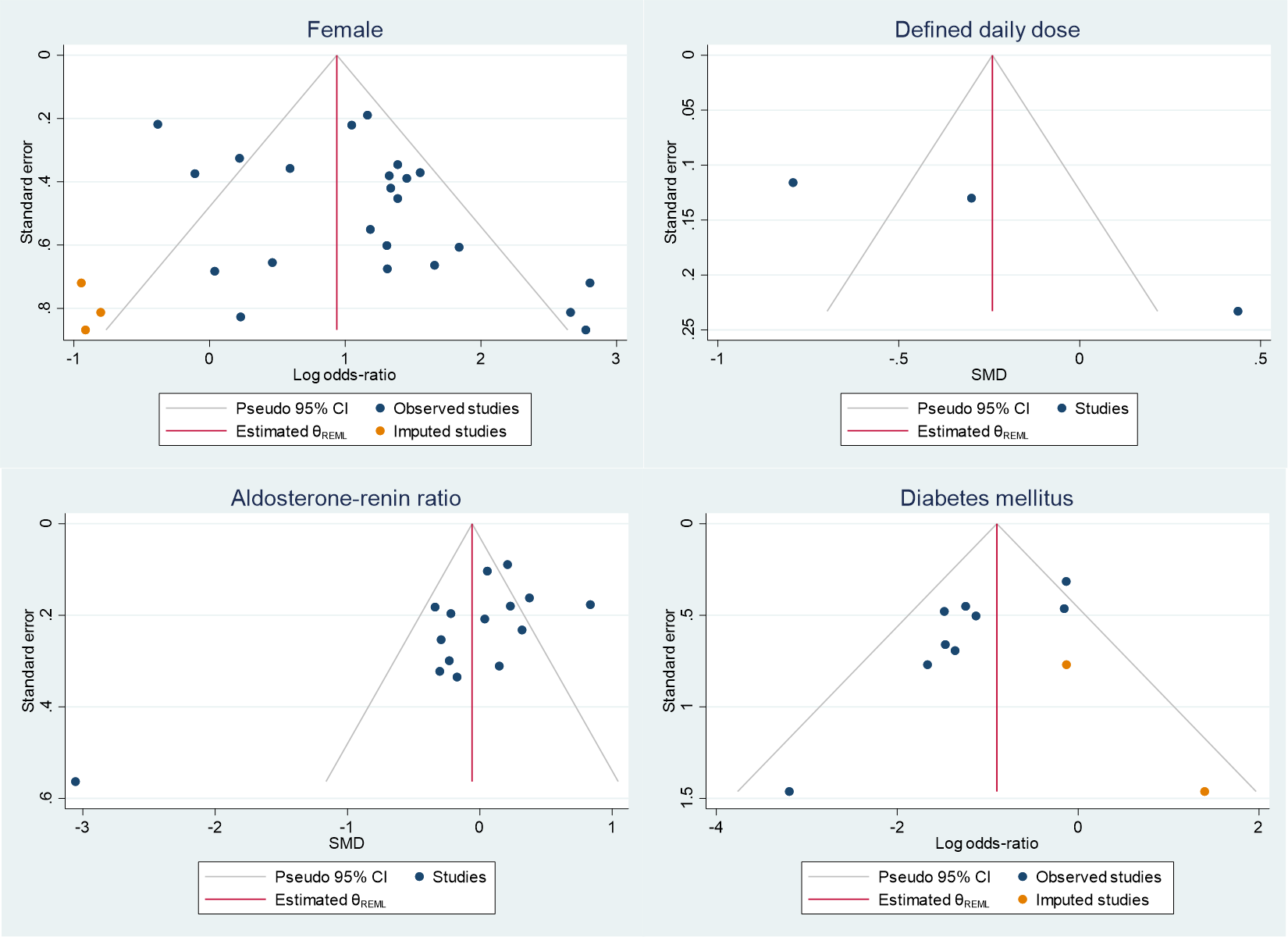
**

**Figure S6.** Funnel plot with trim-and-fill method of predictive factors using crude data
